# Onco-miR-130 promotes cell proliferation and migration by targeting TGFβR2 in gastric cancer

**DOI:** 10.18632/oncotarget.9936

**Published:** 2016-06-10

**Authors:** Jingjing Duan, Haiyang Zhang, Yanjun Qu, Ting Deng, Dingzhi Huang, Rui Liu, Le Zhang, Ming Bai, Likun Zhou, Guoguang Ying, Yi Ba

**Affiliations:** ^1^ Department of Gastrointestinal Oncology, Tianjin Medical University Cancer Institute and Hospital, National Clinical Research Center for Cancer, Tianjin Key Laboratory of Cancer Prevention and Therapy, Tianjin, 300060, China

**Keywords:** TGFβR2, miR-130, gastric cancer, proliferation, migration

## Abstract

MicroRNAs (miRNAs) have been proved to play crucial roles in tumorigenesis. TGFβ signal pathway abnormality is found in various cancers and correlates with tumor proliferation and metastasis. However, the mechanisms underlying the dys-regulation of TGFβR2 expression in GC have not been investigated yet. In this study, we found that the TGFβR2 protein was clearly repressed in tumor tissues, while miR-130 expression level was dramatically increased in GC tissues. Firefly luciferase activity assay revealed that miR-130 could directly bind to 3′UTR of TGFβR2 mRNA. Meanwhile, miR-130 mimics lead to the decreased TGFβR2 protein levels, while miR-130 inhibitors enhanced TGFβR2 expression in SGC7901 cells. Subsequent functional experiments showed that overexpressed miR-130 could promote proliferation and migration of SGC7901 cells. And siRNA-mediated TGFβR2 down-regulation could simulate the effects of miR-130 mimics on phenotypes of SGC7901 cells. Furthermore, there existed intense relationship between the expression level of miR-130 and epithelial-mesenchymal markers. Our results demonstrated that miR-130 was an oncogene by directly targeting TGFβR2 in GC.

## INTRODUCTION

Gastric cancer (GC) is the fourth most common cancer worldwide [1]. The incidence rate of GC in Asian countries is higher than others, furthermore, China account for 42% in Asia [2]. With its aggressive properties and rather late stage at diagnosis, the prognosis of gastric cancer is still poor. Although great advance has been made in the diagnosis and treatment of GC, it could only improve curative effect of early disease, but not the curative effect in case of middle and late stages. Current treatment, such as chemotherapy and radiation, still cannot significantly prolong the survival time of gastric cancer patients. Targeted medicine, which is based on cancer gene therapy, can be a promising strategy in the treatment of gastric cancer [3]. However, the effective and comprehensive driven target used in treatment is limited in gastric cancer. Hence, many researchers focused on the mechanisms of GC oncogenesis to explore novel targets.

The transforming growth factor beta (TGFβ) signaling pathway is known to be involved in many cellular processes, including cell growth, differentiation, apoptosis and cellular homeostasis [4]. TGFβ1 is a secretory cytokine that binds to the TGFβ-receptor-2 (TGFβR2), which then complexes with the TGFβ-receptor-1 (TGFβR1 or ALK1 or ALK5). This binding complexes activates TGFβ signaling via smad phosphorylation and nuclear translocation [5]. The TGFβ pathway is always aberrant in many diseases including cancers [6–8]. In diverse malignancies, TGFβ signaling has either tumor suppressive or pro-oncogenic functions in accordance with tumor stage [9, 10]. The blockade of TGFβ signaling is always through either interrupt of TGFβ responses or the development of genetic alterations and epigenetic modifications in its components especially TGFβR2 [11]. As a tumor-suppressor gene, TGFβR2 is down-regulated in multiple cancer types [7, 12–14]. Then cancer cells lose their sensitivity to TGFβ-mediated growth inhibitory effect due to TGFβR2 down-regulation [15]. Although the role of TGFβ signaling pathway has been discovered, the mechanisms underlying the down-regulation of TGFβR2 expression especially in the tumorigenesis of GC have not been investigated yet.

MicroRNAs (miRNAs) are a class of small non-coding single-stranded RNA molecules of approximately 22 nucleotides, which are involved in a wide spectrum of biological processes including development [16], aging process [17], cell proliferation [8], cell apoptosis and the immune response [18]. MiRNAs lead to specific mRNA cleavage or translational repression through forming miRNA-induced silencing complexes and binding to the 3′-untranslated region (3′UTR) of target gene mRNA [19]. Abnormal expression of miRNAs act as essential modulators for carcinogenesis, chemotherapy resistance and tumor metastasis [19]. Recent years, serum miRNAs are regarded as novel biomarkers for the diagnosis and prognosis of various cancers [20, 21]. The properties that miRNAs are highly conserved in the genome and have a relatively high degree of tissue specificity make miRNAs ideal biomarkers for cancer identification. Our previous studies even show that miRNAs can be secreted into the extracellular environment through microvesicles (MVs) and function as secretory signaling molecules that influence the recipient cell phenotypes [22].

MiR-130 has been identified in modulating the porcine reproductive and respiratory syndrome virus (PRRSV) replication [23] and involved in matrix remodeling in pulmonary hypertension [24]. However, the role of miR-130 in tumorigenesis, especially in gastric cancer, has not been investigated yet. In our present study, we found that miR-130 is up-regulated, whileas TGFβR2 expression is significantly suppressed in GC tissues. The subsequent luciferase assay showed that miR-130 can directly bind to the 3′UTR of the mRNA of TGFβR2. The inhibition of TGFβR2 in GC cells which was resulted from the overexpression of miR-130, promoted cell proliferation and migration. Therefore, our data illustrated that the novel miR-130-TGFβR2 pathway play a key role in the tumorigenesis of GC, which could act as a potential target in the future treatment of GC.

## RESULTS

### TGFβR2 is down-regulated in human gastric cancer tissues

**Figure 1:**
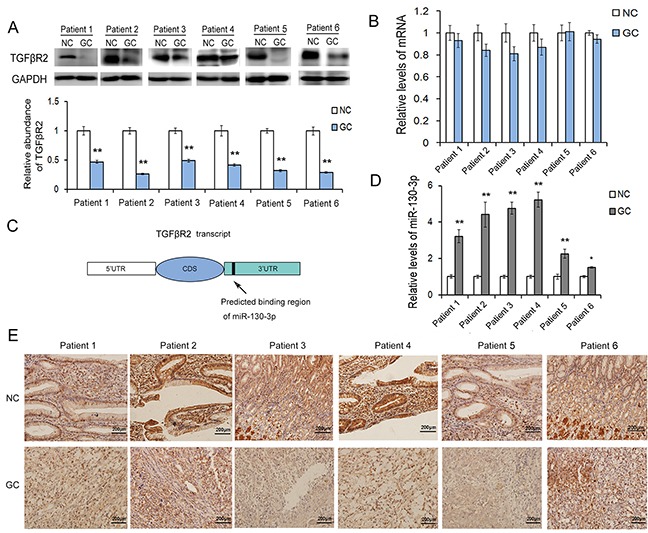
The expression patterns of TGFβR2 and miR-130 in GC tissues **A.** Western blot analysis of TGFβR2 expression in GC cancer tissues and the paired adjacent noncancerous tissues (n=6). **B.** Relative levels of TGFβR2 mRNA levels in GC tissues (n=6). **C.** The predicted binding region of miR-130 in the mRNA of TGFβR2. **D.** Relative levels of miR-130 in GC tissues and para-carcinoma tissues (n=6). **E.** Immunohistochemistry assays of TGFβR2 expression in GC cancer tissues (n=6). * indicates *P*<0.05;** indicates *P*<0.01.

In order to discuss the role of TGFβR2 in gastric carcinogenesis, expression of TGFβR2 was detected by western blotting and qRT-PCR analyses. Six pairs of GC patient tissues and corresponding noncancerous tissues were collected in our study. Among those patients, there were 4 men and 2 women. The average age was 59 (range, 44-69). And all patients received radical gastrectomy without any complication. As shown in Figure 1A, the TGFβR2 protein was found to be significantly down-regulated in GC tissues. However, the mRNA level of TGFβR2 showed only slight decrease in GC tissues compared to the noncancerous tissues (Figure 1B). The expression characteristic of TGFβR2 was detected by using IHC assays in six pairs of formalin-fxed, paraffn-embedded sections of tissue specimens. TGFβR2 is a well-known transmembrane protein, as shown in Figure 1E, it is found to be mainly expressed in cell membrane, and however, due to strong positive of cell membrane in some epithelium cells, it also appeared weak positive in cytoplasm. The gland epithelium of gastric mucosa expressed most TGFβR2. In accordance with the results of western blotting, TGFβR2 is lowly expressed in the sections of cancer tissue specimens compared to corresponding noncancerous tissues. These results suggested that the expression of TGFβR2 mainly depends on the post-transcriptional regulation in GC.

### MiR-130 is up-regulated in GC tissues and directly targets TGFβR2

**Figure 2:**
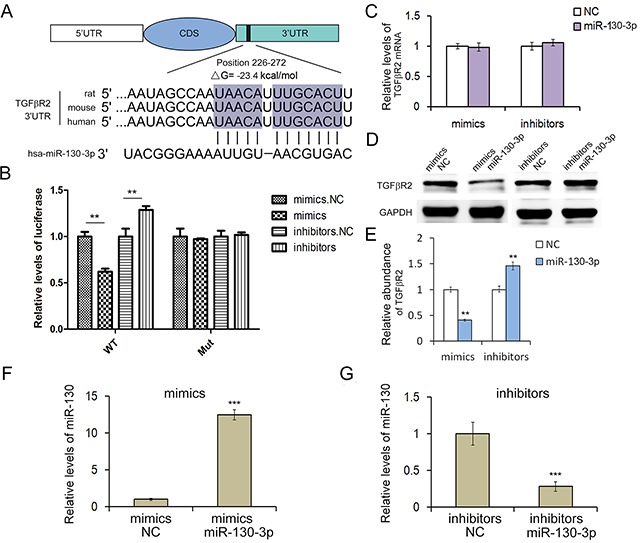
MiR-130 regulates TGFβR2 expression in gastric cancer cells **A.** The predicted base-pairing interaction between miR-130 and TGFβR2 mRNA. **B.** Direct recognition of TGFβR2 by miR-130. SGC7901 cells were co-transfected with firefly luciferase reporters containing either WT or mutant TGFβR2 3′UTR with miR-130 mimics and inhibitors (n=3). **C.** Quantitative RT-PCR analysis of TGFβR2 mRNA levels in SGC7901 cells transfected with miR-130 mimics or inhibitors (n=3). **D.** The suppression of TGFβR2 expression by miR-130 in SGC7901 cells (n=3). **E.** Quantitative analysis of D (n=3). **F.** and **G.** Quantitative RT-PCR analysis of miR-130 levels in SGC7901 cells transfected with mimics or inhibitors (n=3). ** indicates *P*<0.01; *** indicated *P*<0.001.

The miRNA-mediated specific mRNA cleavage or translational repression is one of the most important post-transcriptional regulatory mechanisms. In our study, we use three computational approaches to predict the potential target of miR-130. Based on the predictive results of bioinformatics tools, we find that miR-130 can directly target the 3′UTR of TGFβR2 mRNA, and the free energy of all three computational algorithms show that miR-130 have high affinity with TGFβR2. As shown in Figure 2A, the position that miR-130 directly targets is highly conserved, and miR-130 could binds with TGFβR2 mRNA by complementary base pairing of this target region. To validate the actual relationship of miR-130 and TGFβR2 in GC, we firstly explored the level of miR-130 in six pairs of GC tissues and corresponding noncancerous tissues. As is expected, miR-130 showed obvious increase in all the tumor tissues (Figure 1D). The levels of miR-130 and TGFβR2 showed inverse correlation in GC. Therefore, miR-130 is most likely to be the important regulator of TGFβR2 in gastric cancer cells.

Then we conducted luciferase assay to investigate the direct interaction between miR-130 and TGFβR2. In order to verify this targeting relationship, the miR-130 binding sequence in the 3′-UTR of TGFβR2 and the mutated 3′-UTR sequence were inserted into the downstream of the firefly luciferase reporter gene in p-MIR vector and then co-transfected with miR-130 mimics, inhibitors (or miRNA NC) into HEK293T cells and SGC7901 cells. As shown in Figure 2B, the relative luciferase activity of the reporter gene in SGC7901 cells co-transfected with p-MIR-TGFβR2 and miR-130 mimics was significantly decreased by nearly 50% compared with the control (co-transfected with p-MIR-TGFβR2 and miRNA NC), while the relative luciferase activity of the reporter gene in SGC7901 cells co-transfected with mutated p-MIR-TGFβR2 and miR-130 mimics or miRNA NC was no different. The luciferase signal showed relative increase when miR-130 inhibitors were used instead. The results in HEK293T cells were shown in Supplementary Figure S1, the outcome was consistence with that in SGC7901 cells. Our results verified that miR-130 could suppress TGFβR2 expression through miR-130 binding sequences at the 3′UTR of TGFβR2 mRNA.

### MiR-130 regulates TGFβR2 expression in SGC7901 cells

In order to clarify the function of miR-130, miR-130 mimics or inhibitors were transfected into SGC7901 cell line. After 24h, the cells were collected for detecting miR-130 levels by qRT-PCR analysis. Results showed that miR-130 mimics could significantly increase miR-130 level in SGC7901 cells (Figure 2F), while miR-130 inhibitors decreased miR-130 level (Figure 2G). To further investigate if miR-130 affected TGFβR2 expression at post-transcriptional level in GC cells, we performed qRT-PCR and Western blot to determine the mRNA and protein level of TGFβR2 after the transfection of miR-130 mimics or inhibitors in SGC7901 cells. As shown in Figure 2C and 2D, the overexpression of miR-130 by transfection of mimics led to the clear suppression of TGFβR2 protein, but not TGFβR2 mRNA. While the transfection of miR-130 inhibitors enhances the expression of TGFβR2 in SGC7901 cells. Meanwhile, TGFβR2 mRNA was not changed with the transfection of inhibitors. These data demonstrated that miR-130 is an important post-transcriptional regulator of TGFβR2 in GC cells.

### MiR-130 promotes cell proliferation and migration in SGC7901 cells

**Figure 3:**
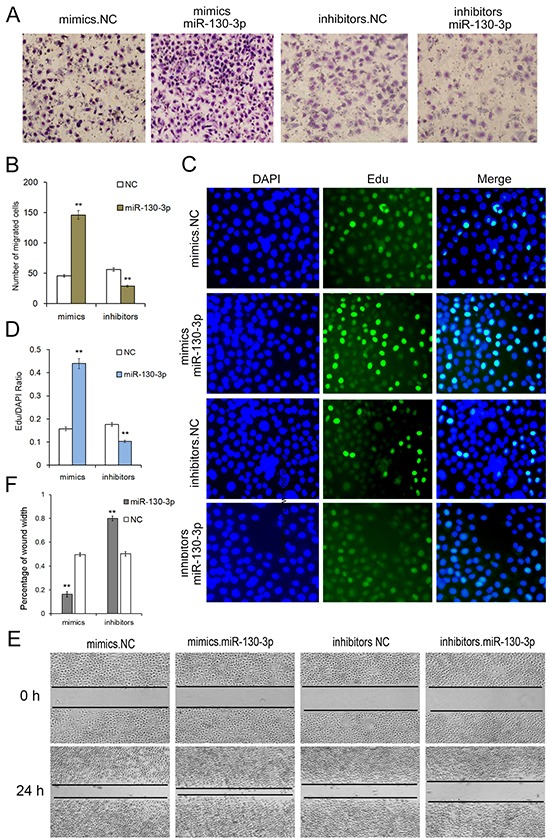
Onco-miR-130 promotes cell proliferation and migration of SGC7901 cells **A.** Transwell assays show that miR-130 promotes cell migration of GC cells (n=3). **B.** Quantitative analysis of A (n=3). **C.** MiR-130 enhances cell proliferation of SGC7901 cells (n=3). **D.** Quantitative analysis of C (n=3). **E.** Validation of miR-130-mediated cell migration by wound healing method (n=3). ** indicates *P*<0.01.

The effect of miR-130 on the proliferation of SGC7901 cells was detected in vitro by the EdU proliferation assay (Figure 3C). The results revealed that the proliferation rate in SGC7901 cells transfected with miR-130 mimics was significantly increased compared with the control group. While, the proliferation rate in SGC7901 cells transfected with miR-130 inhibitors showed a sharp decrease than the NC inhibitor group. All these data showed that miR-130 could enhance the proliferation of SGC7901 cell line.

In order to determine whether miR-130 affected cell migration, wound healing method (Figure 3E) and transwell assay (Figure 3A) were performed after transfection with miR-130 mimics, inhibitors or miRNA NC in SGC7901 cell line. Both the wound healing method and transwell assay demonstrated that SGC7901 cells transfected with miR-130 mimics showed a higher ratio in migration. On the contrary, cell migration was strongly inhibited when cells are transfected with miR-130 inhibitors. These results showed that miR-130 is an onco-miRNA in gastric cancer that could enhance proliferation and migration, and plays a key role in the tumorigenesis in cancer cells.

### Effects of TGFβR2 overexpressing and silencing in SGC7901 cells

**Figure 4:**
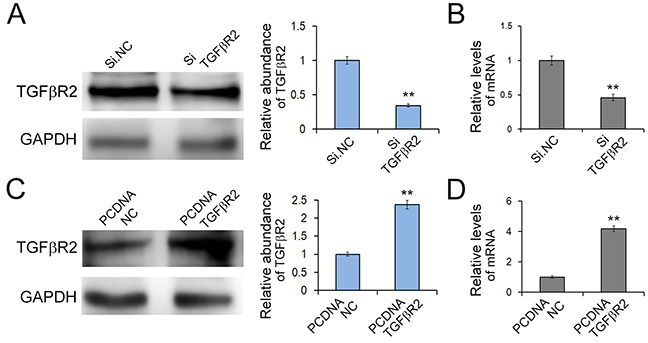
Effects of TGFβR2 silencing and overexpressing in SGC7901 cells **A.** and **B**. Knock-down of TGFβR2 expression by siRNA. SGC7901 cells were transfected with TGFβR2 siRNA, and the protein levels (A) and mRNA levels (B) were detected respectively. (n=3) **C.** and **D.** Up-regulation of TGFβR2 expression by plasmid. SGC7901 cells were transfected with TGFβR2 plasmid, and the protein levels (C) and mRNA levels (D) were detected respectively. PCDNA TGFβR2 refers to overexpression plasmid, and PCDNA NC refers to control plasmid. (n=3) ** indicates *P*<0.01.

**Figure 5:**
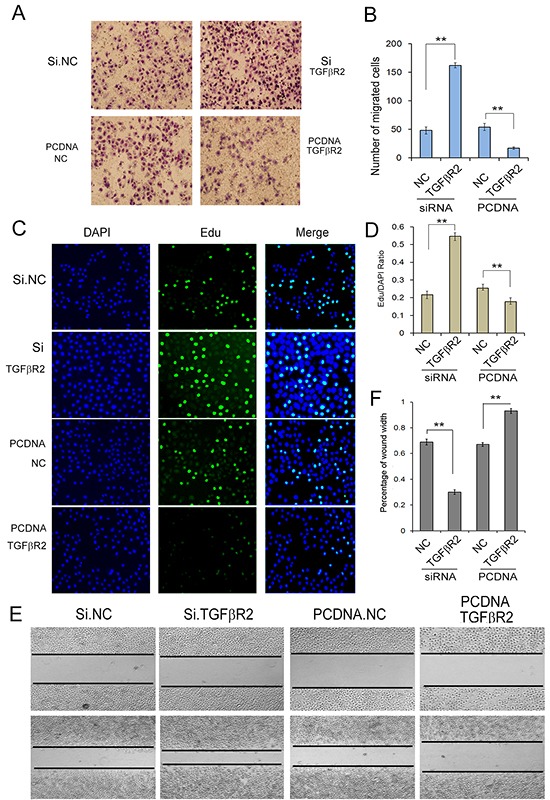
Identification of TGFβR2 as a tumor suppressor in GC **A.** Transwell assays show that knock-down of TGFβR2 enhances cell migration of GC cells strongly (n=3). **B.** Quantitative analysis of A (n=3). **C.** Edu assays demonstrate that knock-down of TGFβR2 promotes cell proliferation of SGC7901 cells (n=3). **D.** Quantitative analysis of C (n=3). **E.** Validation of TGFβR2-mediated cell migration by wound healing method. PCDNA TGFβR2 refers to overexpression plasmid, and PCDNA NC refers to control plasmid. (n=3). ** indicates *P*<0.01.

To further understand TGFβR2-regulated cell growth and migration in GC cells, we used plasmid to overexpress TGFβR2 and siRNA targeting TGFβR2 to down-regulate its expression. Firstly we explored the appropriate concentration of siRNA, and it was shown that the concentration of 100 pmol was appropriate (Supplementary Figure S2). As shown in Figure 4, the expression levels of TGFβR2 mRNA and protein were significantly up-regulated or down-regulated in SGC7901 cells transfected with TGFβR2 plasmid or siRNA compared with control. Subsequently, the function of TGFβR2 was also measured. The EdU proliferation assay (Figure 5C) indicated that the proliferation rate of SGC7901 cells transfected with TGFβR2 plasmid was significantly lower than that of control cells, whereas siRNA targeting TGFβR2 could promote proliferation. Furthermore, the down-regulation of TGFβR2 could promote cell migration significantly (Figure 5A and 5E). These results demonstrated that siRNA-mediated TGFβR2 down-regulation could mimic the effects of miR-130 up-regulation in GC cells. Therefore, TGFβR2 acts as a cancer suppresser in GC and its dramatic down-regulation contributes to faster rate of proliferation and stronger ability of migration in cancer cells.

### The correlation of miR-130-TGFβR2 pathway and epithelial-mesenchymal markers

**Figure 6:**
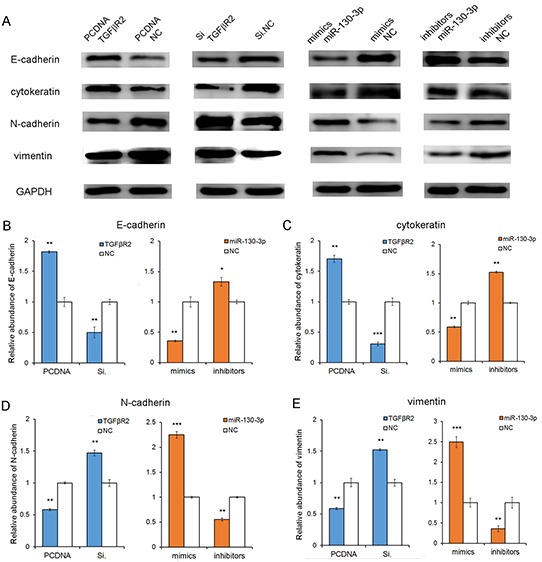
The correlation of miR-130-TGFβR2 axis and epithelial-mesenchymal markers **A.** The results showed that the epithelial markers (E-cadherin and cytokeratin) were up-regulated when miR-130 was knockdown or TGFβR2 was overexpressed. While, it showed a mesenchymal phenotype when miR-130 was up-regulated or TGFβR2 was inhibited. **B, C, D** and **E**. Quantitative analyses of A. PCDNA TGFβR2 refers to overexpression plasmid, and PCDNA NC refers to control plasmid. * indicates *P*<0.05; ** indicates *P*<0.01; *** indicated *P*<0.001.

To further investigate the role of the miR-130-TGFβR2 pathway in gastric cancer cells, we checked for epithelial and mesenchymal markers by western blot analysis. As shown in Figure 6, it showed a mesenchymal phenotype after miR-130 overexpression or TGFβR2 knock-down; whereas an epithelial phenotype when the expression level of miR-130 was inhibited or TGFβR2 was up-regulated. The results emphasis the oncogenic role of miR-130 in GC cells.

## DISCUSSION

Previous studies have clearly indicated that one miRNA could target multiple genes, while one gene could also be regulated by more than one miRNA. For example, miR-24 can target Bim [25] and FSCN1 [26], and Bim can both be mediated by miR-24 and miR-181b [27]. Therefore, identification and perfect the pathway connecting miRNA to their target genes will be helpful to highlight the functions of miRNA in various biological processes containing carcinogenesis.

In present study, we found that the TGFβR2 protein was clearly repressed in tumor tissues, while miR-130 expression level was dramatically increased in GC tissues. TGFβR2 protein expression level was inversely correlated with miR-130 expression level in GC tissues, however, the mRNA level of TGFβR2 showed only slight decrease in tumor tissues. Firefly luciferase activity assay revealed that miR-130 mimics could inhibit luciferase activity in the wild-type 3′UTR of TGFβR2 mRNA but did not work in the mutant construct. Furthermore, miR-130 mimics lead to the decreased TGFβR2 protein levels, while miR-130 inhibitors enhanced TGFβR2 expression in SGC7901 cells. Subsequent functional experiments showed that overexpressed miR-130 could promote proliferation and migration of SGC7901 cells. And siRNA-mediated TGFβR2 down-regulation could simulate the effects of miR-130 mimics on phenotypes of SGC7901 cells, while plasmid-mediated TGFβR2 overexpression had the identical effects with miR-130 inhibitors. All these data verified that miR-130 was an oncogene by directly targeting TGFβR2 which functions as a tumor suppressor in GC. Finally, the relationship of miR-130 and epithelial-mesenchymal markers emphasis the oncogenic role of miR-130. We believed that the secretion of onco-miR-130 from gastric cancer cells is reduced, resulting in the up-regulation of miR-130 in cancer cells. However, this needs more evidence in further studies. Since we performed all experiments in only one gastric cancer cell line SGC7901, the effectiveness of miR-130 for GC in general needs more investigation.

TGFβ pathway is important in many life process including tumor development, but the role of TGFβ signaling pathway in tumorigenesis is controversial. Many papers demonstrated that TGFβR2 functioned as a tumor suppressor and was down-regulated in some human cancers. Low levels of TGFβR2 proteins were negatively correlated with poor survival in patients with nasopharyngeal carcinoma [13]. However, some researchers found that TGFβR2 as a proto-oncogene, the expression was significantly higher in NSCLC tissues than in non-neoplastic tissues, and high expression of TGFβR2 was a critical risk factor for reduced survival in NSCLC patients [7]. And in subsequently vitro experiments, TGFβR2 could promote malignant characteristics in A549 [28] and H1299 cells [29]. However, the role of TGFβR2 in GC tumorigenesis is unknown. Dys-regulation of miRNA expression in various cancers has been widely reported in both tumor tissues and serum [21, 30, 31]. Tissue miRNAs are highly conserved in the genome and could provide an accurate diagnosis for various types of malignancies, which usually lead to disorders of protein expression in cancer cells. To our knowledge, this is the first attempt to investigate the miR130-TGFβR2 pathway in GC tissues and cells. Our results show that miR-130 act as an oncogenic miRNA in GC.

Currently, chemotherapy and radiotherapy are the two major viable strategies exist for the treatment of advanced GC. But resistance to chemotherapeutic drugs has become increasingly problematic in GC. It drives urgent need to detect new targets and treatment strategies. MiRNAs play a vital role in tumorigenesis, angiogenesis and drug resistance [32–34], and are proved to be a useful biomarker in the diagnosis and prognosis of cancer. Recently, miRNAs or anti-miRNAs trafficked by microvesicles (MV) or other plasmids have been used for the treatment of various diseases in mouse models, including cancers [22, 35, 36]. The translational research on miRNAs, including miR-130, will bring a new chapter in cancer gene therapy.

In conclusion, the miR-130-TGFβR2 pathway is involved in the processes of cell growth and migration, thus regulating tumorigenesis in GC. Our study provided further evidence of the extensive role of miR-130 in the regulation of GC progression. Further studies are needed to explore its clinical application as potential molecular therapeutic target for human GC.

## MATERIALS AND METHODS

### Human tissue

Gastric cancer tissues and their paired adjacent noncancerous tissues were derived from patients undergoing a radical surgery at the Tianjin Medical University Cancer Institute and Hospital. Both tumor tissues were histopathologically verified adenocarcinoma and noncancerous tissues were confirmed negative. The study was approved by the the Ethics Committee of Tianjin Medical University Cancer Institute and Hospital and informed consent was obtained before surgery. Tissue fragments were immediately frozen in liquid nitrogen at the time of surgery. Total protein and RNA of those paired tissues were extracted and stored at −80°C.

### Cell culture

Human gastric cell line SGC7901, human embryo kidney epithelial cell line HEK293T were cultured in RPIM1640 (Gibco, USA) or DMEM (Gibco, USA), which was supplemented with 10% fetal bovine serum (FBS, Gibco, USA) and 1% penicillin–streptomycin in a humidified incubator at 37°C with 5% CO_2_ followed by suggestions.

### The miRNA target prediction and Luciferase reporter assay

The miRNA target prediction and analysis were performed with the algorithms from TargetScan (http://www.targetscan.org/), PicTar (http://pictar.mdc-berlin.de/) and miRanda (http://www.microrna.org/). The reporter plasmid p-MIR-TGFβR2 containing the predicted miR-130 targeting regions was designed by Genescript (Nanjing, China). Part of the wild type and mutated 3′UTR of TGFβR2 was cloned immediately downstream of the firefly luciferase reporter. The β-galactosidase expression vector (Ambion) was used as a transfection control. For the subsequent luciferase reporter assays, 2 mg of firefly luciferase reporter plasmid, 2 mg of β-galactosidase vector, and equal doses (200 pmol) of mimics, inhibitors, or scrambled negative control RNA were transfected into the prepared cells. At 24 h after transfection, cells were analyzed using the Dual Luciferase Assay Kit (Promega) according to the manufacturer's instructions. Each sample was prepared in triplicate, and the entire experiment was repeated three times.

### Cell transfection

SGC7901 cells were plated in six-well or other plates and performed transfection after 24 hours. The TGFβR2 overexpressing plasmid (PCDNA TGFβR2) and the control plasmid (PCDNA NC) were bought from GenePharma (Shanghai, China), and 2μg of plasmid were transfected into every single well. Cells were transfected with scrambled negative control, miR-130 mimics or inhibitors using lipofectamine 2000 (Invitrogen, Life Technologies) and Opti-MEM Reduced Serum Medium (Gibco, Life Technologies) according to the manufacturer's instructions. And equal amounts (100 pmol) of miRNA mimics, inhibitors, siRNAs (Santa Cruz), or scrambled negative control RNA were transfected into each well. Then the cells were harvested at 24 h after transfection for real-time quantitative PCR analysis and at 48h for western blotting.

### RT-PCR

Total RNA was extracted from the cultured cells and tissues using TRIzol Reagent (Invitrogen) following the manufacturer's protocol. The quantity of miRNA was performed using Taqman microRNA probes (Applied Biosystems, Foster City, CA). After the reactions were accomplished, the cycle threshold (CT) data were determined using fixed threshold settings, and the mean CT was calculated from triplicate PCRs. A comparative CT method was used to compare each condition to the control reactions. U6 snRNA was used as an internal control of miRNAs, and the mRNA levels of TGFβR2 was normalized to the corresponding housekeeping gene GAPDH. The relative amount of gene normalized to control was calculated with the equation 2^−ΔCT^, in which ΔCT = CT _gene_ − CT _control_. All of the reactions were run in triplicate. Primers of TGFβR2 and GAPDH were as follows:

5′-AGAAGGCTGGGGCTCATTTG-3′ (GAPDH, sense);5′-AGGGGCCATCCACAGTCTTC-3′ (GAPDH, antisense);

5′-GTAGCTCTGATGAGTGCAATGAC-3′ (TGFβR2, sense);

5′-CAGATATGGCAACTCCCAGTG-3′ (TGFβR2, antisense).

### Western blotting

The TGFβR2 expression was assessed by western blotting analysis and samples were normalized to GAPDH. Total protein were extracted from the cultured cells and tissues were solubilized in lysis buffer. The protein were separated by sodium dodecyl sulfate–polyacrylamide gel electrophoresis and then transferred to polyvinylidene difluoride membranes (Roche). The membranes were blocked within 2% Bovine Serum Albumin (BSA) at room temperature for 1 h and incubated overnight at 4°C with primary anti-TGFβR2 (1:200, Santa Cruz), and anti-GAPDH (1:5000, Santa Cruz), respectively. The membranes subsequently washed and incubated with appropriate secondary antibodies. After incubated with ECL, the protein bands were visualized.

### Cell proliferation assay

The proliferative ability of SGC7901 cells after different transfection were determined by the EdU proliferation assay (RiboBio Inc.). Twenty-four hours after transfection, cells were incubated in 50 M EdU for 5 h, and fixed within 4% paraformaldehyde for 30 min at room temperature (RT). Then the cells were washed in PBS twice and permeabilized using PBS containing 0.5% Triton X-100 for 10 min. After extensive washes in PBS, the cells were incubated lucifugally in Apollo staining solution (RiboBio Inc.) for 30 min, then repeated permeation and wash, and incubated in Hoechst 33342 (1:100; RiboBio Inc.) for another 30 min at RT. All of the staining were performed in triplicate.

### Cell migration assay

Wound healing assay and transwell-chamber (Corning, New York, USA) migration assay were used to determine the migrative capacity of transfected cells. When the cells attached, a wound healing assay was performed. After washing with PBS, medium with 1% FBS was added. Then, at the indicated time points of 0 hour, 6 hours, 18 hours and 24 hours, cells were observed and photographs were taken. The distance of the wound zone in each well was measured at least three randomly selected fields and compared with control. After 24h of post-transfection, cells were transferred into the upper chamber of the transwell with 200μl serum-free growth medium (10^5^ cells per well of 8.0 μm Pore Polycarbonate Membrane Insert). Complete medium containing 10% FBS was added to the lower chamber as a chemo-attractant. After 24h of incubation at 37°C, nonmigratory cells on the upper surface of upper chamber were removed slightly by cotton swabs, and cells that migrated to the bottom of the membrane were fixed and stained. The number of invaded cells was counted under light microscope. To minimize the bias, five randomly selected fields with 200× magnification were counted, then the average number was calculated.

### Immunohistochemistry assays

Formalin-fxed, paraffn-embedded sections of tissue specimens including gastric adenocarcinoma and paired adjacent noncancerous tissues were reviewed by pathologists. TGFβR2-examinations in tissues were performed on 8-μm-thick paraffin sections. All sections were deparaffnized twice with xylene and rehydrated in a graded series of ethanol. The sections were soaked in 10 mmol/L citrate buffer (pH 6.0) for antigen retrieval, and heated to 220°C in high pressure for 3 minutes. Endogenous peroxidase activity was blocked by soaking in 3% hydrogen peroxide for 20 minutes. The sections were then incubated overnight at 4°C with anti-human TGFβR2 monoclonal antibody (1:50, Santa Cruz, sc-400). The next day, the slides were washed in PBS and incubated with second antibodies for 40 minutes at 37°C. After washes with PBS, 3-amino-9-ethylcarbazole solution were used to chromogen. Then the sections were counterstained with hematoxylin, dehydrated, and coverslipped. Quantitative analysis was conducted by quantifying the fluorescence intensity from six sections.

### Statistical analyses

All statistical analyses were performed using IBM SPSS Statistics, Version 20.0. All data were representative of at least three independent experiments. Data were described with median values ± SME and analyzed by using the Student's t test for 2-group comparisons. Differences were considered statistically significant for *P*<0.05. In this study, ‘*’ indicates ‘*P*<0.05′, ‘**’ indicates ‘*P*<0.01′, and ‘***’ indicates ‘*P*<0.001′.

## SUPPLEMENTARY FIGURES


