# Supplementary material for: Onco-miR-130 promotes cell proliferation and migration by targeting TGFβR2 in gastric cancer
Source: Oncotarget. 2016 Jun 10;7(28):44522–33. doi: 10.18632/oncotarget.9936 (PMC5190115; doi:10.18632/oncotarget.9936)
Supplement: Supplementary file 1 [file oncotarget-07-44522-s001.pdf]

Onco-miR-130 promotes cell proliferation and migration by targeting TGFβR2 in gastric cancer

SUPPLEMENTARY FIGURES

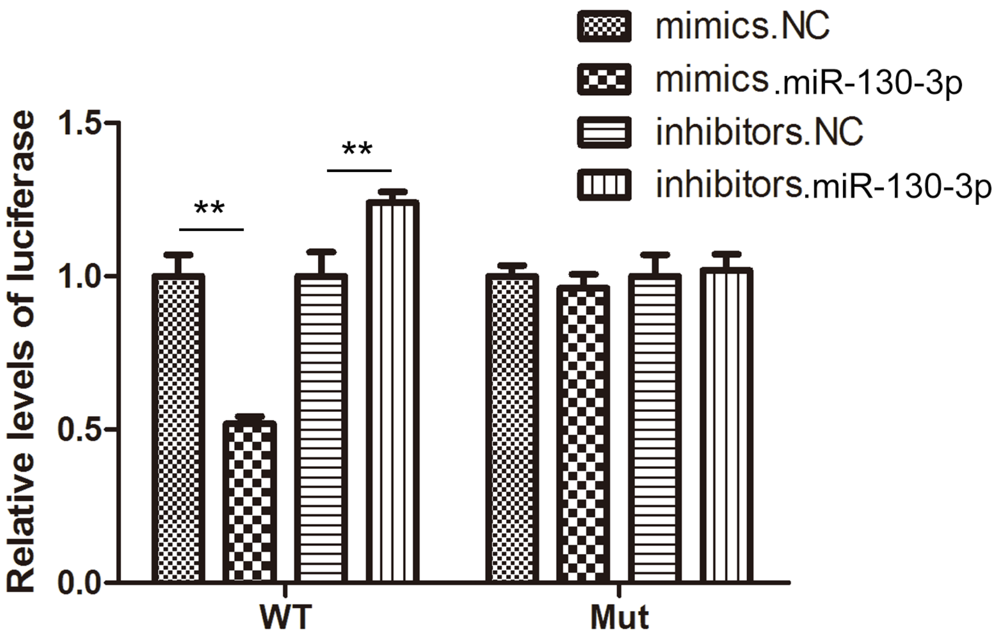

Supplementary Figure S1: The luciferase reporter gene analysis in HEK293T cells. The results show that miR-130 can directly bind to the 3′UTR of TGFβR2. ** indicates *P*<0.01.

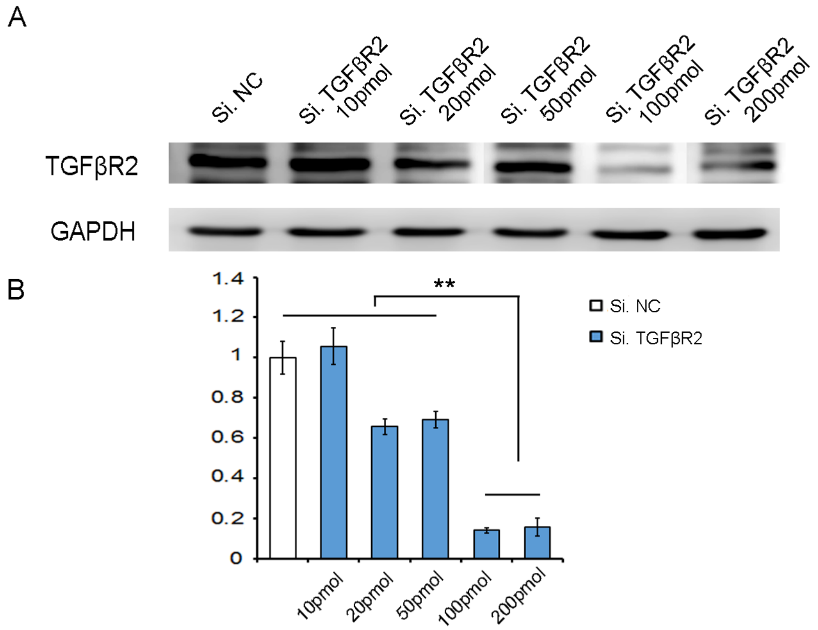

Supplementary Figure S2: The western blotting in siRNA-TGFβR2 treated cells from low concentration to high concentration. It is proved that the concentration of 100 pmol is appropriate. **A.** The western blotting analysis. **B.** Quantitative analyses of A. ** indicates *P*<0.01.
